# Tuning the mechanical properties and degradation properties of polydioxanone isothermal annealing

**DOI:** 10.3906/kim-2006-55

**Published:** 2020-10-26

**Authors:** Xiliang LIU, Shaomin FENG, Xin WANG, Jin QI, Dong LEI, Yadong LI, Wei BAI

**Affiliations:** 1 Chengdu Institute of Organic Chemistry, Chinese Academy of Sciences, Chengdu P.R. China; 2 University of Chinese Academy of Sciences, Beijing P.R. China

**Keywords:** Polydioxanone (PPDO), isothermal annealing, mechanical properties, hydrolytic degradation in vitro

## Abstract

Polydioxanone (PPDO) is synthesized by ring-opening polymerization of p-dioxanone, using stannous octoate as the catalyst. The polarized optical micrograph (POM) shows thes pherulite growth rate of PPDO decreases with an increase in the isothermal crystallization temperature. PPDO is compression-molded into bars, and PPDO bars are subjected to isothermal annealing at a range of temperatures (Ta = 50, 60, 70, 80, 90, and 100 °C), and correspond to three different annealing times (ta = 1h, 2h, 3h). The effect on PPDO is investigated by using differential scanning calorimetry (DSC), thermogravimetric analysis (TGA), X-ray diffraction (XRD), and scanning electron microscopy (SEM). With an increase in Ta and ta, the grain size and the degree of crystallinity also increase. Meanwhile, the tensile strength is significantly improved. The PPDO bars (90 °C, 2 h) reach the maximum crystallinity (57.21%) and the maximum tensile strength (41.1 MPa). Interestingly, the heat treatment process does not result in serious thermal degradation. It is observed that the hydrolytic degradation of the annealed PPDO is delayed to some extent. Thus, annealed PPDO might have potential applications, particularly in the fields of orthopedic fixation and tissue engineering.

## 1. Introduction

With the rapid development of agriculture, medicine, and food packaging, people are increasingly using polymer materials. Petro-chemical-based synthetic polymers (polyethylene, polypropylene, polystyrene, etc.) are difficult to degrade in the natural environment [1]. These polymer materials seriously pollute the environment and endanger living organisms. As environmental awareness has increased in recent decades, the biodegradable materials offered in different areas have attracted increasing interest [2,3].

In order to avoid the possible risks of metal implants implanted in the human body, such as bone corrosion, stress shielding effect, defects of nondegradation, and the need for secondary surgery, absorbable polymer materials are being increasingly favored by researchers. At present, absorbable polymers are mostly used for implant materials that do not require high strength. Besides, the degradation properties are also one of the important factors affecting the application of absorbable polymers.

Polydioxanone (PPDO) is a unique aliphatic polyester, synthesized by ring-opening polymerization of p-dioxanone. PPDO is similar to polylactic acid (PLA), polyglycolic acid (PGA), and polycaprolactone (PCL), etc., whose main molecular chain contains ester bonds, endowing polymers with excellent biodegradability, biocompatibility, and bioabsorbability [4]. Furthermore, due to the unique ether bond, PPDO has excellent flexibility as an ideal medical biodegradable material [5]. The degradation products produced by PPDO are less acidic than PGA and PLA [6]. PPDO is one of the few polymers that have been approved by the Food and Drug Administration (FDA). PPDO as a long term surgical suture (e.g., PDS II by Ethicon) provides support during healing periods longer than four weeks [7]. In addition to its successful application in the surgical suture, PPDO is used to make orthopedic fixation materials, tissue repair materials, cell scaffolds, and drug carriers [8,9]. Since the polymer is easy to be processed by injection-molding, PPDO also has great potential for general use, such as in films, molded products, laminates, foams, nonwoven materials, adhesives, and coatings [10]. However, the mechanical strength of PPDO is relatively low, which hinders its use in applications requiring high mechanical strength. Besides, compared to other polyester polymers, PPDO degrades much faster. In a case where PPDO scaffolds are implanted in the subcutaneous tissue of mice, almost complete degradation is observed after 28 days [11]. Atrial septal defect (ASD) is one of the most common congenital heart defects, and it accounts for about 30% of the congenital heart diseases. PPDO as ASD occluders degrades gradually over about 24 weeks in an acute animal (canine) model [12].

Heat treatment has been proven to affect the crystallization and mechanical properties of materials [13–15]. The possibility of improving the mechanical properties of PPDO via thermal treatment has been proposed in this context [16,17]. Zhao et al. [18] have improved the compressive performance of PPDO self-expandable stents through thermal treatment conditions (60 ℃, 80 ℃, and 100 ℃, 1 h). At the same time, the heat treatment process might damage the mechanical properties of PPDO because of thermal degradation or thermal oxygen degradation. Li et al. [19] give some details about the thermal stability of PPDO by thermogravimetric (TG) analysis. Nishida et al. [20,21] have studied the thermal decomposition of PPDO in nitrogen and discussed the thermal decomposition mechanism. Therefore, the selection of heat treatment conditions is very important.

The isothermal annealing is essential to adjust the final physical properties of the material in the course of processing [22–24]. The semicrystalline nature of PPDO makes it possible to adjust the final physical properties of the applications through isothermal annealing. Obtaining optimum properties for future polymer devices produced by PPDO would require an understanding of the annealing process. From a safety perspective, the tensile strength is beneficial for the design of medical implants in the future, such as surgical sutures, orthopedic fixation, etc. However, to our knowledge, there is still a lack of a clear relationship between heat treatment and the properties of PPDO. To guide the design and manufacture, we prepared the PPDO samples via compression molding and investigated the effects of annealing temperature (Ta) and annealing time (ta) on the mechanical properties and degradation properties.

## 2. Materials and methods

### 2.1. Materials

According to the references [25–27], PPDO ( *M*_v_ = 1.13×10 ^5^ g·mol ^-1^ ) was synthesized by ring-opening polymerization of the para-dioxanone monomer under vacuum at 120 °C for three days, using stannous octoate as the catalyst. The polymers were purified by being dissolved in dichloromethane and precipitation into ethanol, washed with fresh ethanol and dried at room temperature under vacuum. The intrinsic viscosity ([η] = 2.43 g·dL ^-1^ ) was measured by Ubbelohde viscometer at 25 ℃, using hexafluoroisopropanol (HFIP, Suzhou Highfine Biotech Co., Ltd., Suzhou, Jiangsu, China) as the solvent. The viscosity-average molecular weight ( *M* v) of PPDO was calculated using the Mark–Houwink equation.

(1)[η]=KMνα

where α is 0.69, and K is 79 × 10 ^-3^ cm ^3^ ·g ^-1^ .

KH _2_ PO _4_ was purchased from Chengdu Chron Chemical Co., Ltd. (Chengdu, Sichuan, China), and Na _2_ HPO _4_ ·12H _2_ O was purchased from Guangdong Guanghua Sci-Tech Co., Ltd. (Guangzhou, Guangdong, China). The other solvents were of analytical grade.

### 2.2. Preparation and isothermal annealing of PPDO bars

PPDO was compression-molded into bars with dimensions in accordance with ASTM standard D638-039 specifications, using a Model XLB platen vulcanizing press (Haimen Jinma Rubber & Plastics Machinery Technology Co., Ltd., Haimen, Hainan, China) at 140℃, and the processing pressure was 12.5 MPa.

The isothermal annealing of PPDO bars [13,18]: Firstly, the vacuum oven (Shanghai Shenxian Instrument Co., Ltd., Shanghai, China) was heated to the designated temperature (50 ℃, 60 ℃, 70 ℃, 80 ℃, 90 ℃, and 100 ℃). A thermometer was placed in the oven to rectify the temperature. At the same time, anhydrous calcium chloride was added for drying. Then, the bars were placed between two sheets of clean glass slide to prevent the material from thermal deformation. Next, the PPDO bars were placed in the vacuum oven. Finally, the oven was evacuated to prevent the thermal oxygen degradation of the bars. When the time was over (1 h, 2 h, 3 h), the oven stopped heating, and the bars were taken out after being cooled to room temperature.

### 2.3. Characterization

#### 2.3.1. Polarized optical micrograph (POM)

The spherulite growth of the sample was observed using a polarized optical microscope (XPN-203) equipped with a stage temperature controller [XMT-3000A(4000A)]. The samples were first heated to 150 °C, and they were held at this temperature for 3 min to be pressed into film and to destroy thermal history. Then, the samples were cooled to the desired crystallization temperatures (Tc) and held at the Tc for the performing of a spherulite. The polarizing microscopic pictures of the samples were taken by a camera (TK-C921EC, Victor Company of Japan, Ltd., Yokohama, Japan) at regular intervals to calculate the spherulite growth rate.

#### 2.3.2. Tensile testing

The mechanical properties of the PPDO bars were measured using a Model CMT 4503 type SANS tensile tester (Shenzhen Xinsansi Material Testing Co., Ltd., Shenzhen, Guangdong, China) with a drawing speed of 20 mm/min at room temperature according to ASTM standard D638. Each reported value was the mean of five parallel samples.

#### 2.3.3. Differential scanning calorimetry (DSC)

The annealed samples (5–10 mg) were cooled to –40℃ and held for 5 min. Then, the samples were heated to 150 ℃ at a heating rate of 10 ℃/min under nitrogen flow using a Model Q20 DSC (TA Instruments Inc., New Castle, DE, USA).

#### 2.3.4. Thermogravimetric analysis (TGA)

Thermogravimetric analysis (TGA) was carried out with Mettler TGA2 (Mettler-Toledo International Inc., Greifensee, Switzerland) to measure the decomposition temperature of the materials. The specimen (10 mg) was heated from 20 ℃ to 600 ℃ under a nitrogen atmosphere at a heating rate of 10 ℃/min.

#### 2.3.5. X-ray diffraction (XRD)

X-ray diffraction (XRD) was performed with the TTR III XRD (Rigaku Corp., Tokyo, Japan), employing Ni-filtered CoKα as radiation (λ= 1.54 Å) at 48 kV and 100 mA. A scan axis of 2 *θ* (10~35°, 5 °/min)was used to obtain diffraction patterns. The full width at half maximum (FWHM) was obtained from the crystallization peak at 2 *θ* of 21.90°, using the XRD analysis software (Jade Software Corp., Christchurch, New Zealand). *X*_C_ was calculated from the XRD patterns:

(2)XC,WAXD=AcrAcr+Aamx100%

Where *X*_C, WAXD_ , *A* _am, and *A* _cr represent the crystallinity, the areas of amorphous, and crystalline peaks (2 *θ* = 10~35°), respectively. *A* _am and *A* _cr are evaluated by the curve fitting method [17].

The Debye–Scherrer formula reflected the correspondence between grain size and FWHM:

(3)DKγBcosθx100%

where D is the average thickness of the crystal grains perpendicular to the crystal plane direction. K is the Scherrer constant, and γ is the X-ray wavelength (1.54 Å). B is the half-height width of the diffraction peak of the measured sample. *θ* is the Bragg diffraction angle.

### 2.4. Degradation properties

#### 2.4.1. Degradation experiment in vitro

1.65 g of KH _2_ PO _4_ and 19.53 g of Na _2_ HPO _4_ ·12H _2_ O were dissolved in deionized water (1L) to prepare phosphate buffer saline (PBS, pH = 7.40). The annealed PPDO bars (90 ℃, 2 h) reached the maximum tensile strength. Therefore, untreated PPDO and annealed PPDO bars (90 ℃, 2 h) were selected for hydrolytic degradation in vitro.

The annealed PPDO bars were divided into three groups corresponding to 2W, 4W, and 5W respectively, four parallel samples per set. The three sets of samples were completely immersed in PBS buffer solution (25 ml). The degradation experiment was carried out in a 37 °C water-isolated incubator (GHP-9080, Guangzhou Mecan Trading Co., Ltd., Guangzhou, Guangdong, China), and the test was performed periodically [28]. The untreated PPDO bars were prepared in the same way as the annealed bars. The mass loss rate and water absorption rate were calculated according to the following formula:

(4)Mass Loss=mi-mdmix100%

(5)Water Absorption=mw-mdmix100%

where m _i_ is the initial dry weight before degradation, m _w_ is the wet weight after degradation, and m _d_ is the dry weight after degradation.

#### 2.4.2. Scanning electron microscope

After the surface of the sample was subjected to gold spray treatment, the surface morphology of the material after degradation was observed by an Inspect F50 scanning electron microscope (FEI, Thermo Fisher Scientific Inc., Waltham, MA, USA).

## 3. Results and discussion

### 3.1. POM analysis

**Figure 1:**
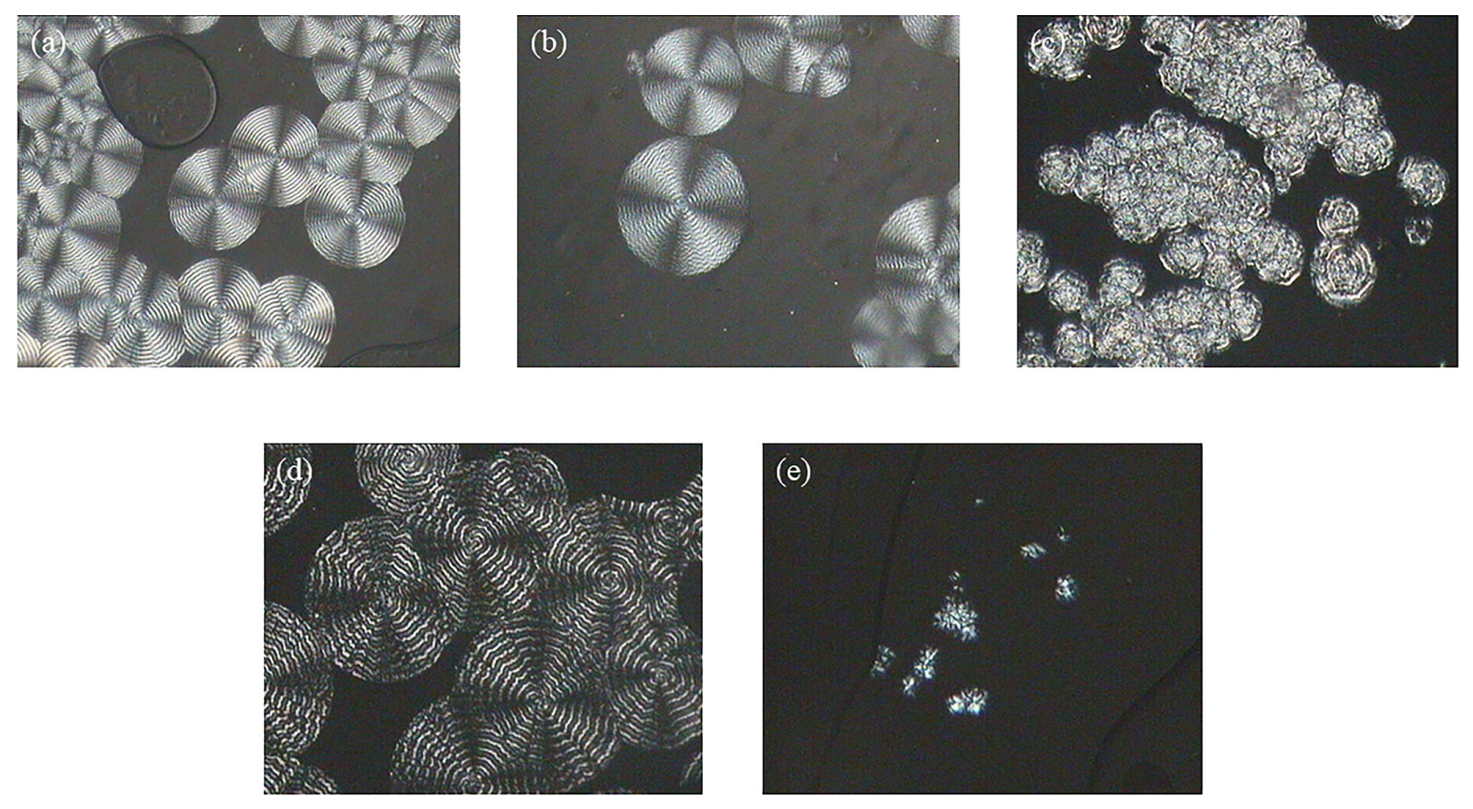
Polarized optical micrographs of PPDO spherulites after isothermal crystallization at different temperatures (Tc) for different time (tc): (a) Tc = 60 °C, tc = 6 min, (b) Tc = 70 °C, tc = 19 min, (c) Tc = 80 °C, tc = 30 min, (d) Tc = 90 °C, tc = 110 min, (e) Tc = 100 °C, tc = 100 min.

Firstly, the growth behavior of PPDO spherulite was studied by POM. Figure 1 showed the polarized optical micrographs of PPDO spherulite after isothermal crystallization at different temperatures. The crystallization was too fast at 50℃ to observe the growth of the spherulite. The spherulites that formed at lower crystallization temperature (Tc=60~90 ℃) showed obvious Maltese cross patterns and concentric rings (Figures 1a–1d) [7,29]. With the temperature increase, the Maltese cross patterns and concentric rings gradually disappeared in Figure 1e. It was replaced by a cracked crystal with a rough surface while the temperature was 100 ℃. This was due to the acceleration of molecular motion, resulting in the irregular growth of spherulites. At crystallization temperatures of 80 ℃ or higher, double banding became very clear since the two different periodicities kept increasing with Tc, as indicated in Figure 1d [30].

**Figure 2:**
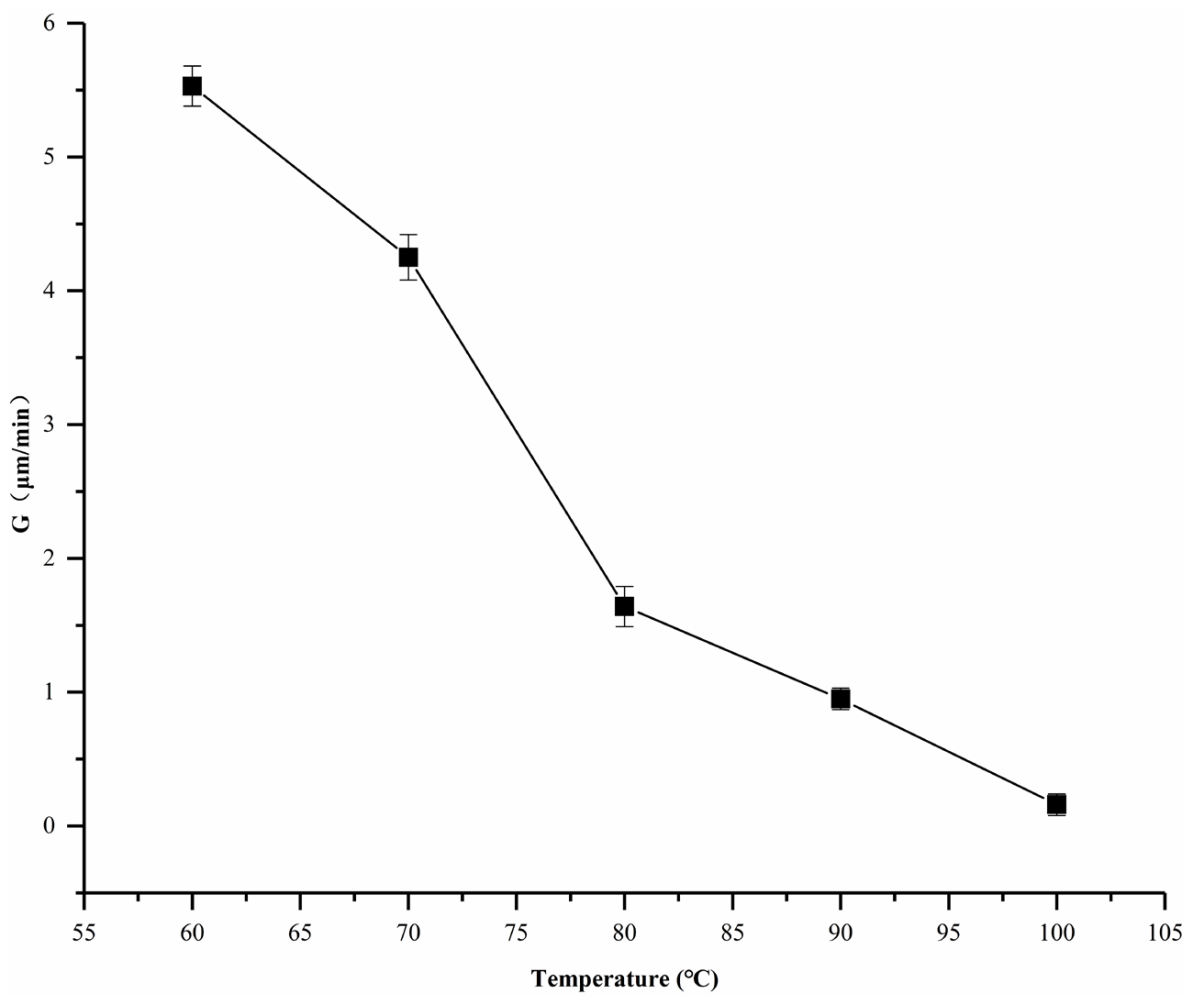
Radius growth rate of spherulites (G) of PPDO as a function of crystallization temperature (Tc).

It could be observed that the growth rate of PPDO spherulite was affected by Tc. The radius growth rate of spherulites (G) was plotted in Figure 2 as a function of Tc. The G value was estimated from the slopes of lines, in which spherulite radii were plotted as a function of crystallization time [13]. It could be seen from Figure 2 that the spherulite growth rate decreased with an increase in Tc. On the one hand, as the temperature went up, the thermal movement of the molecules became more intense. The nuclei which were not easy to form or not stable enough were easily destroyed by molecular thermal movement. As a result, the occurrence time of spherulites at high temperatures was much slower than that at low temperatures. On the other hand, when the temperature approached the melting point, the crystal nucleus was more unstable. Thus, the growth rate of the spherulite was slower. When the temperature was 100 °C, it took a long time for the PPDO spherulite to grow. The POM showed that the spherulite growth rate of PPDO decreased with an increase in Tc.

### 3.2. Mechanical properties

**Figure 3:**
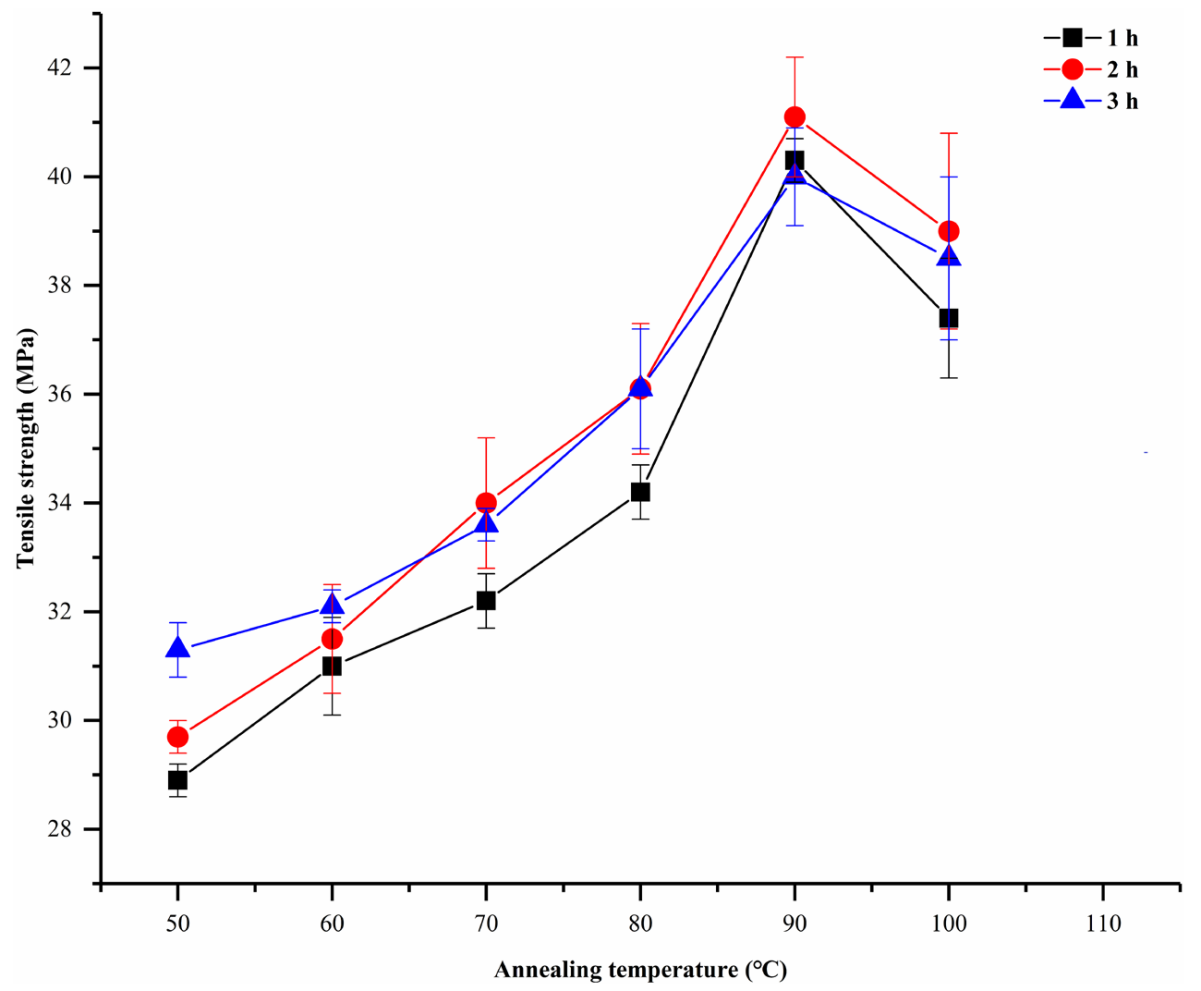
Tensile properties of PPDO bars after isothermal annealing at different Ta.

If the polymer were heated to a temperature above Tg and below Tm, annealing would occur. In practical applications, this could eventually affect the mechanical properties. Tsuji et al. [31] used three different processes to anneal the PLLA film prepared by solution casting: process A, direct annealing of the as-cast film (film A); process B, melting and annealing (film B); and process C, melting, quenching, and annealing (film C). They found that the spherulite density of film C is larger than that of film A and film B. Higher spherulite nucleation density of film C must have shortened the annealing time required for the completion of overall crystallization and decreased the sphere radius. Meanwhile, Tsuji suggested that process C allows producing PLLA films with different Tm by varying Ta, without the morphology change [31]. Zhao et al. [13] annealed PLLGA85/15 bars using a method similar to process C. In this study, we chose to eliminate the thermal history by reaching the melt temperature of PPDO and then quenching to room temperature (PPDO was compression-molded into bars). Then, PPDO bars were annealed after raising the temperature to the desired annealing temperature. The tensile properties of PPDO bars were improved by the isothermal annealing process. The mechanical and thermodynamic performance data of annealed PPDO are given in Table 1. Each reported value was the mean and standard deviation of five parallel samples. The effect of Ta on the tensile strength of annealed PPDO can be seen in Figure 3. The tensile strength had the same tendency regardless of whether ta was 1 h, 2 h, or 3 h. For example, when ta was 1h, observing the change of tensile strength with Ta, the tensile strength of PPDO annealed at 50 ℃ was 28.9 ± 0.4 MPa. The tensile strength could reach 40.3 ± 0.5 MPa at 90 ℃. When Ta further increased to 100 ℃, the tensile strength decreased to 37.4 ± 1.1 MPa. The process of isothermal annealing could effectively adjust the tensile strength by changing Ta, improving the tensile strength of PPDO. For the same ta, PPDO isothermal annealed at 90 ℃ had the maximum tensile strength.

**Table 1:** Thermal- and mechanical properties of untreated PPDO and annealed PPDO. Values are expressed as mean ± standard deviation, n = 5.

Samples	Ta (°C)	ta (h)	T _LM_ (°C)	ΔH _C(LM)_ (Jg ^–1^ )	T _HM_ (°C)	ΔH _C(HM)_ (Jg ^–1^ )	Elastic modulus (MPa)
Untreated PPDO PPDO	25	0	46.07	0.88	106.52	56.25	254.1 ± 37.3
AnnealedPPDO	50	1	61.91	1.97	106.48	51.84	285.3 ± 9.9
	50	2	61.96	2.87	106.79	55.85	297.8 ± 22.6
	50	3	62.73	2.40	106.53	47.90	296.3 ± 17.9
	60	1	71.28	2.45	106.80	52.21	290.1 ± 21.6
	60	2	70.79	3.39	107.23	56.64	296 ± 9.2
	60	3	73.08	2.60	106.50	58.75	296 ± 30.5
	70	1	81.10	4.14	107.76	57.00	288.3 ± 24.7
	70	2	79.75	4.00	106.27	59.91	294.7 ± 20.4
	70	3	83.06	3.58	106.50	51.00	316.5 ± 41.2
	80	1	88.96	2.79	106.38	47.36	299.5 ± 36.2
	80	2	89.98	3.33	106.42	44.78	300 ± 38.2
	80	3	90.11	4.42	106.56	42.06	321.9 ± 35.2
	90	1	-	-	106.19	64.44	381.6 ± 10.6
	90	2	-	-	107.42	65.12	431.0 ± 22.1
	90	3	-	-	108.65	64.97	375.2 ± 33.7
	100	1	-	-	107.35	67.90	484.9 ± 32.2
	100	2	-	-	111.42	70.03	492.8 ± 47.2
	100	3	-	-	110.66	73.96	490.5 ± 45.9

The mechanical properties of aliphatic polyesters were mainly affected by chemical structure and aggregation structure, crystal morphology, and crystallinity [32]. Polymer crystals usually grew under conditions far from thermodynamic equilibrium. Annealing was a dynamic process in which isothermal annealing accelerated the movement of molecular chains to reduce or remove residual stresses and strains, resulting in a more desirable and stable structure. During the annealing, the amorphous regions became locally ordered, and the arrangement of molecular chains in the crystal became compact and regular. Also, the size and number of the crystal grains increased. Choosing different Ta could result in different recrystallization rates and crystallinity, giving different tensile strength. As a result, although different Ta was selected, compared with untreated PPDO, the tensile strength of PPDO increased in different degrees. Annealed at 90 °C, the tensile strength of PPDO was significantly improved. As Ta gradually approached the melting point of PPDO, the tensile strength of PPDO was damaged. The decrease in tensile strength was due to the limited formation of crystal nuclei, which was detrimental to crystallization, resulting in a decrease in crystallinity. This could be obtained by XRD analysis.

**Figure 4:**
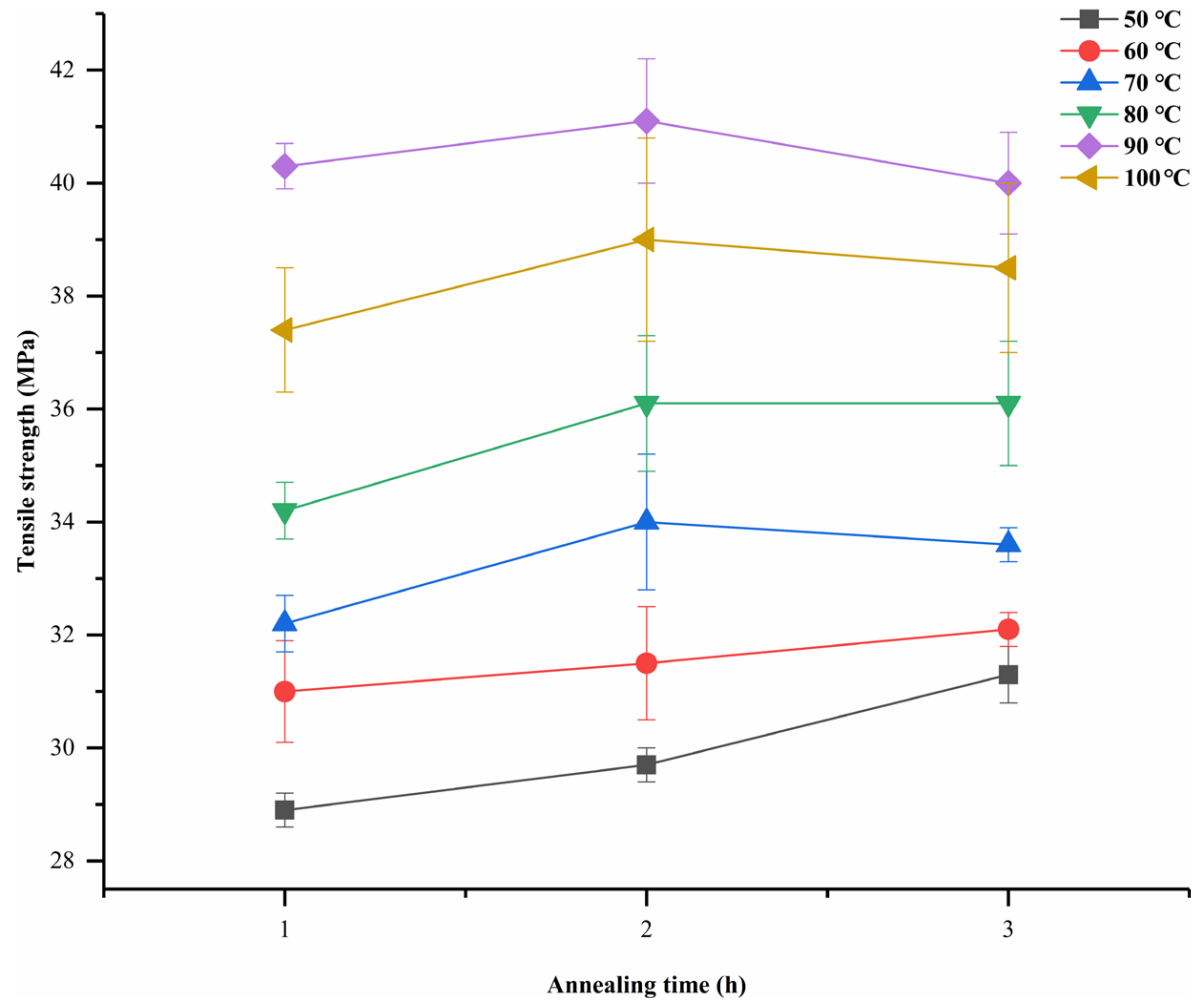
Tensile properties of PPDO bars after isothermal annealing for different ta.

The ta can also affect the tensile strength of PPDO. The effect of ta on the tensile strength of annealed PPDO could be evaluated from Figure 4. The tensile strength had two trends with an increase in ta. One was that at low Ta (50 ℃, 60 ℃), and prolonging the annealing time could increase the tensile strength. Since the diffusion of polymer chains depended on time, the longer annealing time allowed more chains to diffuse into the suitable orientations for crystalline arrangement [33], and the crystal integrity and crystallinity gradually improved. The other was that at high Ta (70 ℃, 80 ℃, 90 ℃, 100 ℃), the tensile strength rose first and then decreased. The full development of the crystalline domains would limit the segmental mobility in the amorphous phase. The transition from the amorphous state to the crystalline state became more difficult. When the crystallization reached a certain level, the crystallinity of the material and the percentage of crystal integrity were almost constant. When ta was 3 h, the tensile strength decreased. The reason for this may be that the material underwent thermal degradation during annealing.

Zhao et al. [18] investigated several thermal treatment conditions (60 °C, 80 °C, and 100 °C for 1 h) to improve the characteristics of PPDO self-expandable stents. They suggested that the 60 °C annealing stents show higher compressive stiffness, and prior viscoelasticity and shape stability are obtained at higher annealing temperatures (80 °C and 100 °C). Thus, the choice of heat treatment conditions was very important. Choosing proper Ta and ta can effectively increase the crystallinity and improve the tensile strength of PPDO. PPDO could reach the maximum tensile strength (41.1MPa)when annealed at 90 ℃ for 2 h. The tensile strength improved by 79.48% compared to untreated PPDO. From a safety perspective, it could provide guidance for the design of tensile products, such as surgical sutures.

### 3.3. DSC analysis

**Figure 5:**
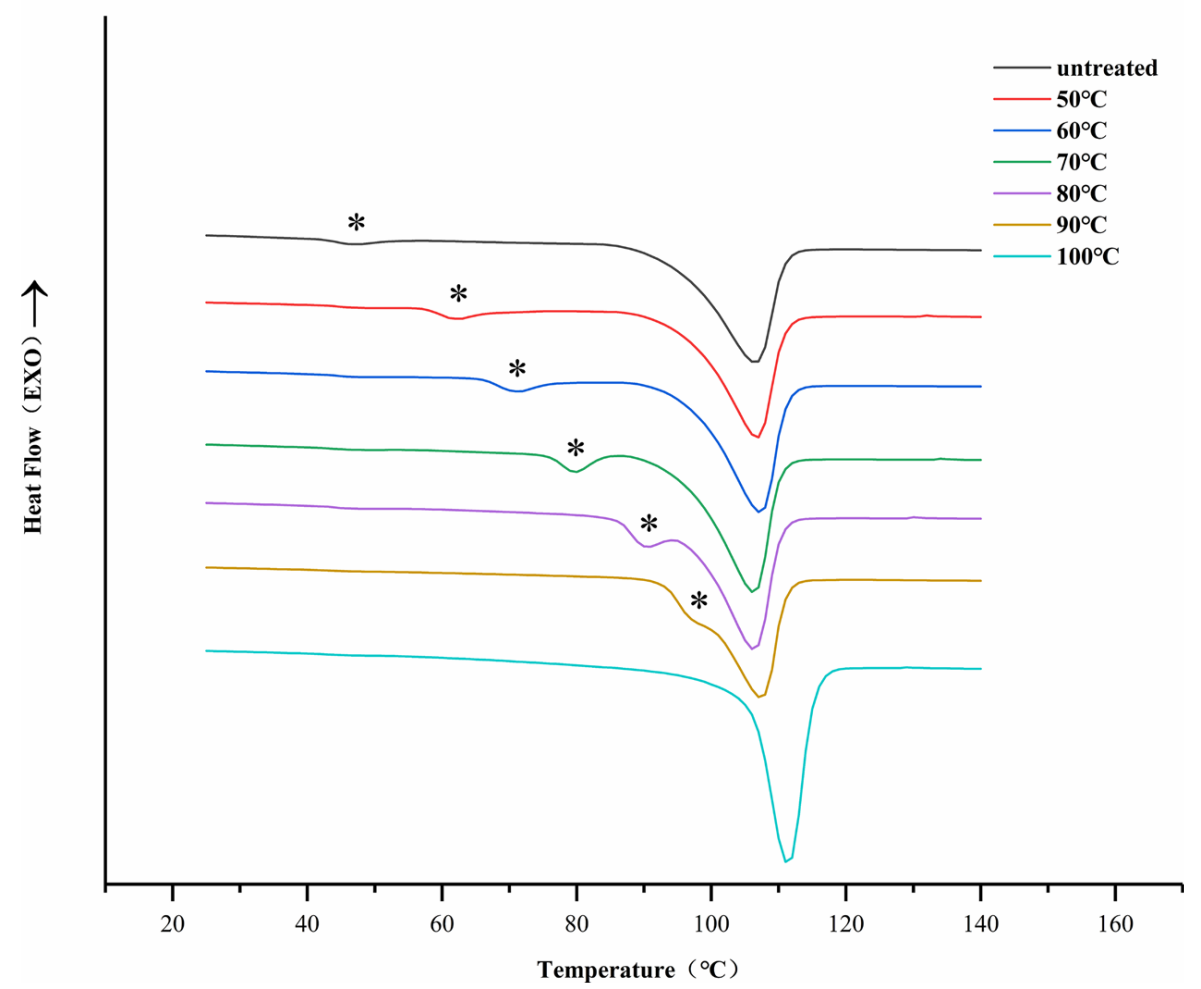
DSC thermograms of PPDO after isothermal annealing at various Ta for 2 h.

**Figure 6:**
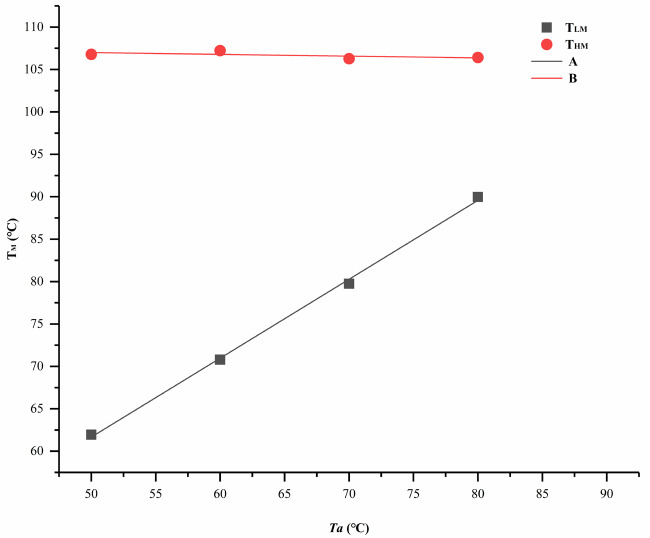
Peak melting temperatures of PPDO as a function of Ta after isothermal annealing for 2 h: (A) linearly fitting of T _LM_ , (B) linearly fitting of T _HM_ .

The DSC heating curves had the same tendency regardless of whether ta was 1 h, 2 h, or 3 h. The DSC curves of PPDO after isothermal annealing for 2 h at various Ta can be seen in Figure 5. It was obvious that the DSC curves showed a baseline shift (asterisk). The melting curve showed two melting endotherms, a weaker lower melting peak (LM), and a higher melting peak (HM) near the melting point [9]. LM moved to a high temperature with an increase in Ta. When Ta≥ 90 ℃, the double melting peak gradually disappeared and turned into a melting peak. The melting points corresponding to the two melting peaks were T _LM_ and T _HM_ , respectively. By linearly fitting the melting point, it could be seen in Figure 6 that the T _LM_ increased linearly. The T _LM_ was approximately 10 °C higher than Ta, indicating the dependence of T _LM_ on Ta. The T _HM_ was stable at 106~108 °C, which was not affected by the change in Ta. After the double melting peak disappeared, the melting point moved to a higher temperature (110 °C).

The annealing process could induce the folding and rearrangement of molecular chains in unstable regions. Therefore, the recrystallization of PPDO occurred during isothermal annealing. Due to the melting of the polymer produced in the secondary crystallization, it could be asserted that the occurrence of the double melting of PPDO was a result of recrystallization. At lower Ta, the initial recrystallization of PPDO produced imperfect crystals. The corresponding T _LM_ at this annealing temperature was also lower. As Ta increased, the less stable regions were melted and redistributed between the more stable regions, and the LM crystals of the double melting peak observed in DSC during heating were reorganized into more ordered HM crystals by recrystallization. The size and perfection of the LM crystal may be increased by the solid-state diffusion mechanism. Therefore, the T _LM_ depended on Ta and was always 10 ℃ higher than Ta. The HM crystals were relatively perfect, so T _HM_ showed independence from Ta. The change in DSC double melting peak and recrystallization can indicate that the annealing process changed the internal structure. Finally, it changed the tensile strength of PPDO.

### 3.4. TGA analysis

**Figure 7:**
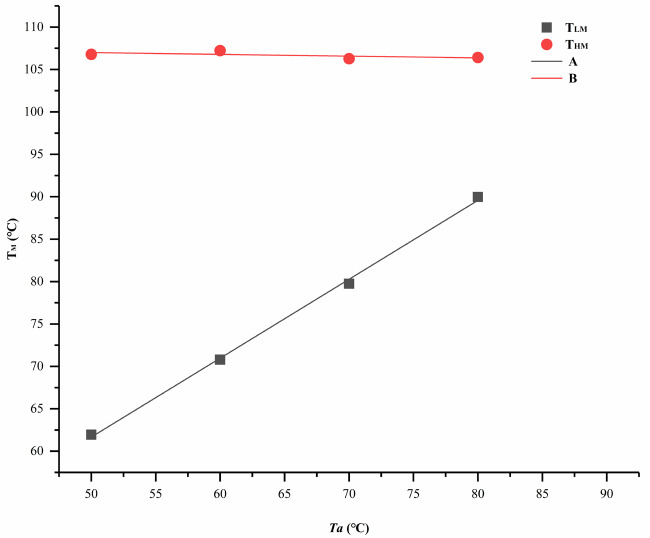
TGA curves of weight loss as a function of temperature for untreated PPDO and annealed PPDO (90 °C, 2h).

The thermal stability of untreated PPDO and annealed PPDO (90℃, 2h) were examined by TGA analyses at a heating rate of 10 ℃/min with a steady flow of nitrogen [19]. The TGA curves are shown in Figure 7, and the data for the samples are summarized in Table 2. The thermal decomposition of PPDO was mainly a zero-order depolymerization process [20,21]. It can be observed that the main thermal degradation step of untreated PPDO started at around 185.25 °C, while this step was initiated at 181.50 °C for annealed PPDO. The temperature at a maximum rate (T _max_ ) of untreated PPDO was about 306.83 °C. However, T _max_ of annealed PPDO appeared at 299.83 °C. Then, the weight of untreated PPDO and annealed PPDO decreased steadily, corresponding to almost complete degradation at 311.83 ℃ and 306.50 ℃, respectively.

When untreated PPDO and annealed PPDO are compared, it can be seen that the main reason for these declines was that PPDO underwent slight thermal degradation during the annealing process, resulting in a decrease in the molecular weight of annealed PPDO. The change in molecular weight can affect the thermal stability of the polymer [34]. As can be seen in Figure 7, annealed PPDO still had good thermal stability. The annealing process did not cause severe thermal degradation of PPDO.

**Table 2:** The TG data of untreated PPDO and annealed PPDO (90 °C, 2h). T _i_ is the initial thermal degradation temperature. T _50%_ is the temperature with weight loss of 50%. T _max_ is the maximum rate temperature. T _d_ is the maximum thermal degradation temperature.

Samples	T _i_ (°C)	T _50%_ (°C)	T _max_ (°C)	T _d_ (°C)
Untreated PPDO	185.25	300.33	306.83	311.83
Annealed PPDO (90 (°C), 2h)	181.50	294.39	299.83	306.50

### 3.5. XRD analysis

**Figure 8:**
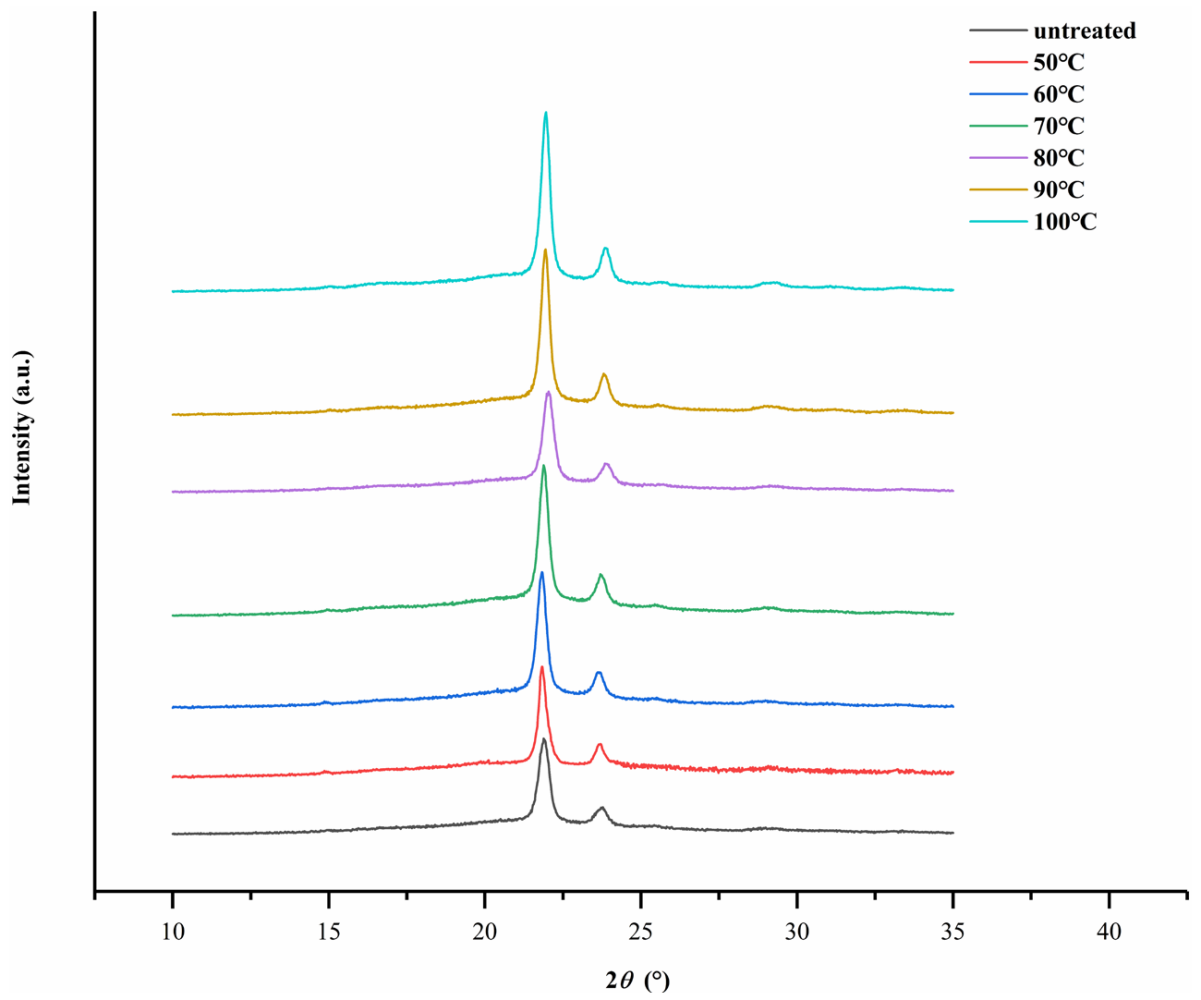
WAXD of PPDO in the range of 10~35° (2-theta) after isothermal annealing at various Ta for 2 h.

The untreated PPDO and annealed PPDO were investigated by XRD. Figure 8 shows the WAXD diagram of PPDO in the range of 10~35° (2 *θ* ) after annealing at various Ta for 2 h. It can be seen that the PPDO has three characteristic diffraction peaks at 21.9°,23.8°, and 29.3°, and the corresponding spacings between the planes in the crystal are 0.405 (d210), 0.373 (d020), and 0.304 (d310) nm, respectively [17]. Compared with untreated PPDO, the annealed PPDO showed little change in the WAXD pattern, indicating that there was no transition of the crystal phase [18]. When Ta ≥ 50 ℃, the main peak became sharper with an increase in Ta. There was not a new diffraction peak formed, and the isothermal annealing did not cause the crystal form change of PPDO.

**Figure 9:**
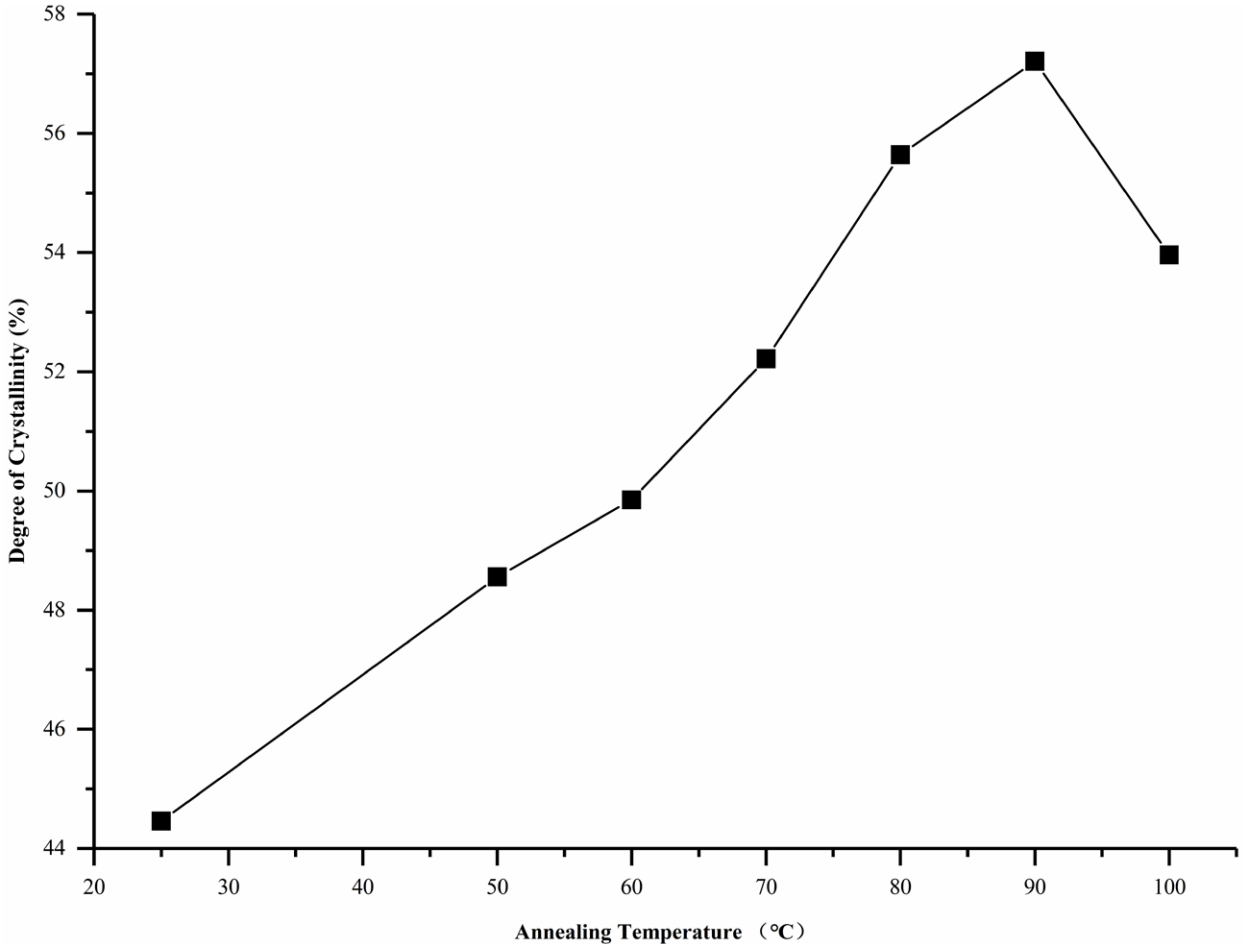
Changes in the degree of crystallinity of PPDO after isothermal annealing at various Ta for 2 h.

The degree of crystallinity was calculated as the percentage of the scattering intensity of the crystalline phase relative to the total scattering intensity of the crystalline phase and the amorphous phase using Eq. (2). PPDO was a semicrystalline polymer whose crystallinity first rose and then decreased with an increase in Ta. The relationship between the crystallinity of annealed PPDO and Ta can be seen in Figure 9. The crystallinity of the untreated PPDO was only 44.46%. When Ta ≤ 70 ℃, the crystallinity smoothly increased. While Ta ≥70 ℃, the crystallinity increased rapidly and reached a maximum (57.21%) at 90 ℃. When Ta further increased to 100 °C, the crystallinity of PPDO decreased and became 53.96%. The crystallinity was one of the main factors that affect the tensile properties of the PPDO. It can be observed that the change in crystallinity was similar to the change in tensile strength. This was consistent with the analysis of the changes in tensile strength that were previously discussed.

**Figure 10:**
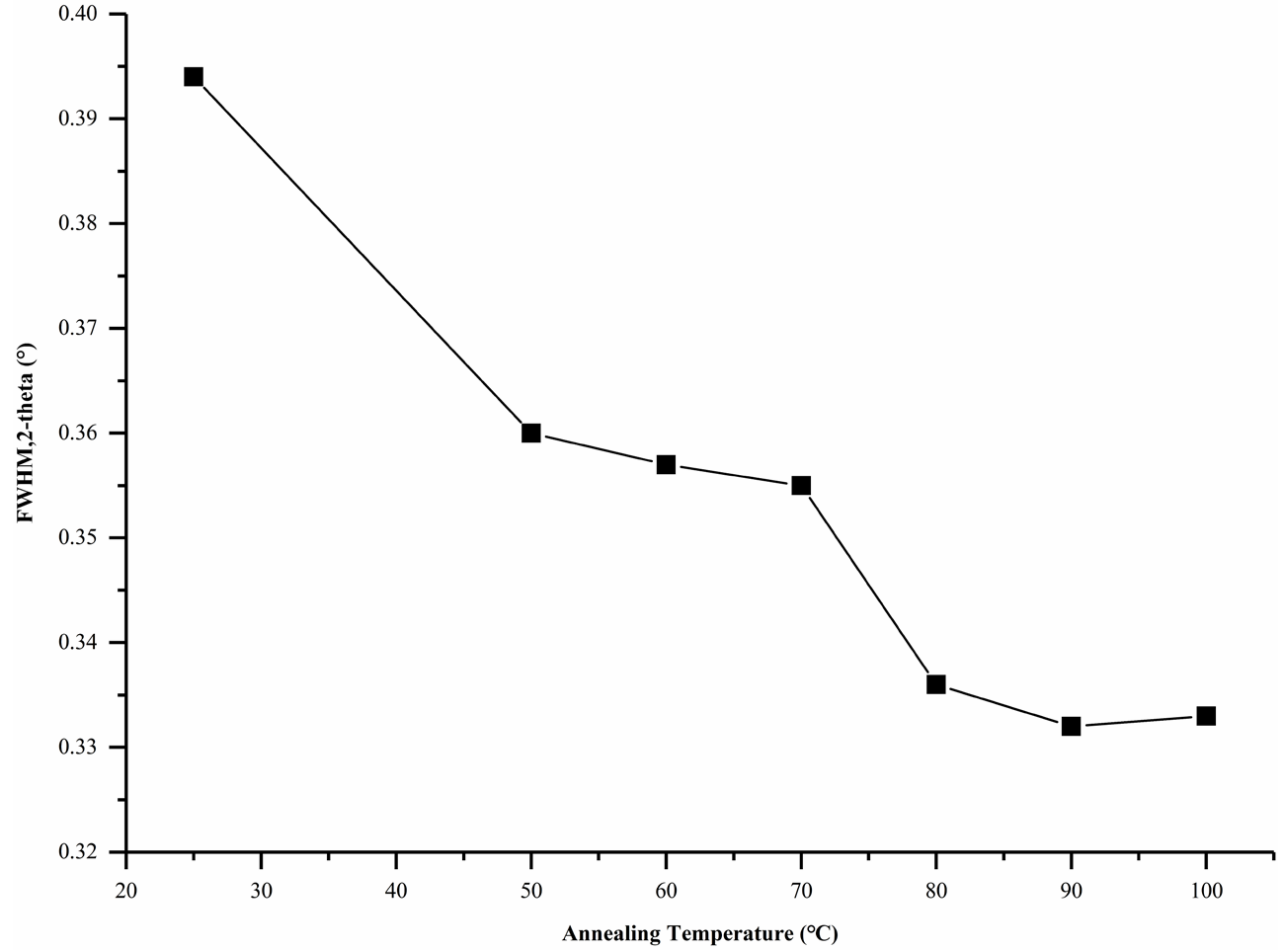
Changes in FWHM of X-ray diffraction peak (21.90°) after isothermal annealing at various Ta for 2 h.

Figure 10 shows the full width at half maximum (FWHM) of the X-ray diffraction peak at 21.90°(2 *θ* ) after isothermal annealing at various Ta for 2 h. Using Eq. (3), the changes of FWHM could be used to roughly represent changes incrystal size [13]. The smaller the FWHM is, the larger the grain size is. Also, the average thickness of the crystal grains in the direction perpendicular to the crystal plane was larger. It could be seen that untreated PPDO had the largest FWHM, which indicated that the grain size of the PPDO was still small at this time. During the isothermal annealing, the FWHM dropped sharply. The FWHM of annealed PPDO (50 °C) decreased from 0.394 to 0.360., and the FWHM reached a minimum at 90 °C of 0.332. It proved that with an increase in Ta, the size of PPDO grains continued to increase and that the crystallization process was more and more perfect. This was consistent with the DSC analysis.

### 3.6. Degradation properties of PPDO

**Figure 11:**
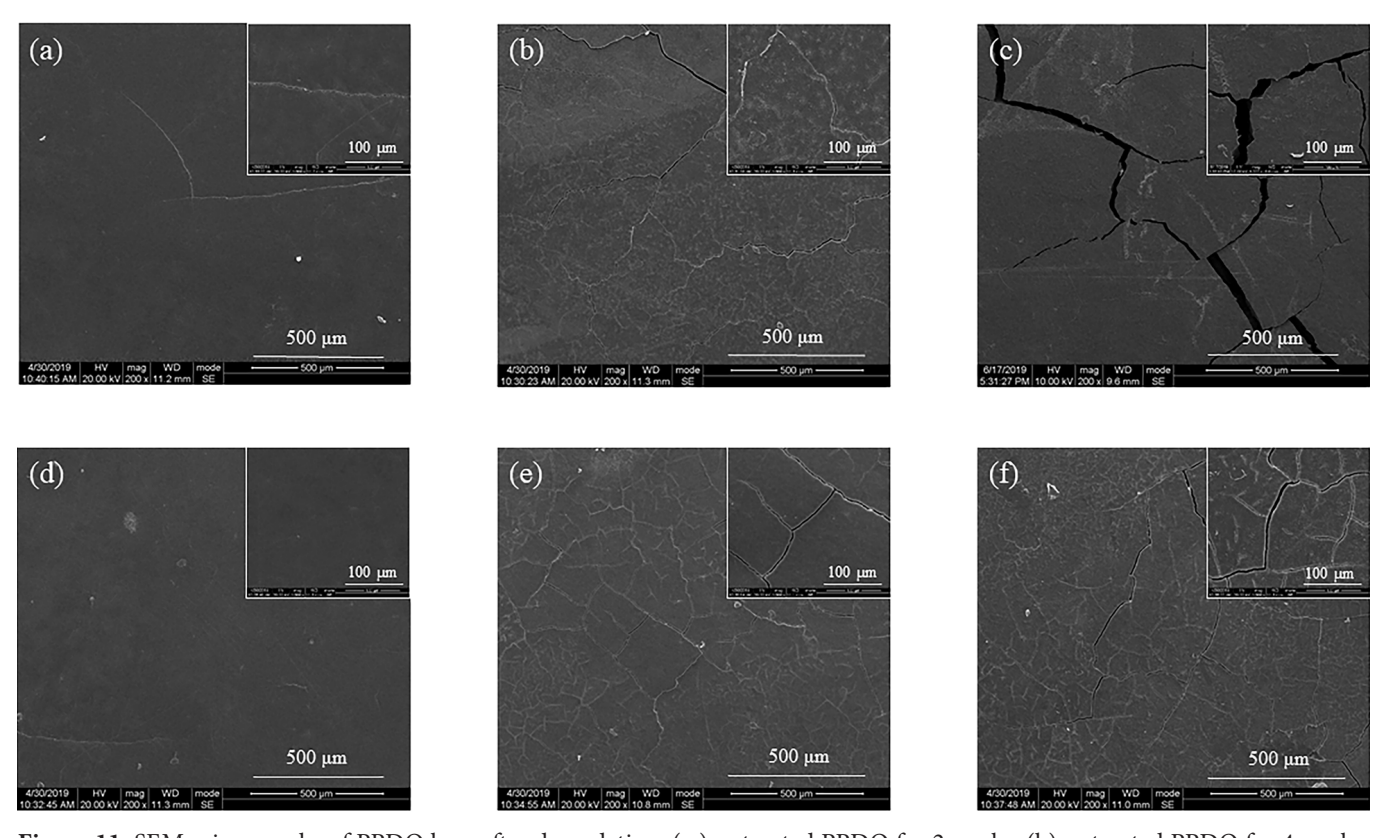
SEM micrographs of PPDO bars after degradation: ( a) untreated PPDO for 2 weeks, (b) untreated PPDO for 4 weeks, (c) untreated PPDO for 5 weeks, (d) annealed PPDO for 2 weeks, (e) annealed PPDO for 4 weeks (f) annealed PPDO for 5 weeks.

Figure 11 shows the morphology of the samples periodically observed by the scanning electron microscope. When the degradation progressed to the 2nd week, the entire surface remained essentially intact without too many defects. As shown in Figure 11a, very few cracks gradually appeared on the surface of the sample. In the 4th week, the surface of the sample changed significantly, as can be seen in Figure 11b. The number of cracks distributed on the surface increased, and the crack size became larger (1–3 μm). Meanwhile, the degraded fragment monomer was exposed on the surface of the substrate in the form of small white particles. This was due to the continuous degradation of the sample under the continuous attack of water molecules [12]. In the 5th week, the cracks developed greatly (4–6 μm), as can be seen in Figure 11c, and the degraded fragments on the surface of the samples also increased obviously. With the progress of degradation, the number of surface defects gradually increased, and the degree of corrosion also gradually deepened.

When the samples were compared among themselves, it was observed that in the 2nd week, there were obvious cracks on the surface, as can be seen in Figure 11a, while the surface of the annealed PPDO was flat as shown in Figure 11d. In the 4th week, when the widest cracks of two sets of samples in Figures 11b and 11e were compared, it was seen that untreated PPDO was larger. Meanwhile, finer cracks were distributed around the widest crack of untreated PPDO, and there was a tendency to further expand. The area around the largest crack of the annealed PPDO retained its original appearance as a whole. In the 5th week, comparing Figures 11c and 11f, we can see that the crack size of untreated PPDO was already significantly larger than that of annealed PPDO and that the size of the fine cracks distributed around the largest crack of the untreated PPDO also became larger. Through the analysis of the surface morphology, it can be found that the surface erosion of untreated PPDO was more serious than that of annealed PPDO. Compared with untreated PPDO, the degradation rate of annealed PPDO was delayed about a week.

The degradation properties in vitro of untreated PPDO and isothermal annealed PPDO (90 ℃, 2 h) were preliminarily evaluated by mass loss, water absorption, and intrinsic viscosity. The degradation performance data of PPDO are given in Table 3. Compared with the untreated PPDO (η = 2.43), the intrinsic viscosity of annealed PPDO (η = 2.26) decreased slightly. The heat treatment process did not cause severe thermal degradation of PPDO. However, the annealed PPDO had a slightly higher intrinsic viscosity during the degradation process. When the degradation reached the 2nd week, the intrinsic viscosity of untreated PPDO decreased by 45.68%, and the intrinsic viscosity of annealed PPDO decreased by 34.51%.

Eqs. (4) and (5) were used to calculate the mass loss and water absorption after the degradation of PPDO. When the degradation reached the 5th week, PPDO bars were not able to maintain the stability of the shape. It was not easy to collect due to the fragmentation of PPDO. Therefore, the degradation data were only recorded until the 4th week. The degradation form of PPDO was mainly hydrolytic degradation. It could be seen in Table 3 that during the whole degradation experiment in vitro, the mass loss and water absorption of untreated PPDO were slightly higher than those of annealed PPDO.

**Table 3:** Degradation properties of untreated PPDO and annealed PPDO (90 (°C),2 h). Values are expressed as mean, n = 4.

Time (weeks)	Samples	0	2	4
Weight retention (%)	Untreated PPDO	100	98.02	92.99
	Annealed PPDO	100	98.29	93.41
Water absorption (%)	Untreated PPDO	0	6.56	11.99
	Annealed PPDO	0	6.31	11.00
Intrinsic viscosity (dL/g)	Untreated PPDO	2.43	1.32	0.56
	Annealed PPDO	2.26	1.48	0.59

As most of the amorphous region hydrolyzed, the hydrolysis medium would diffuse into the crystalline region. If the low molecular weight substances formed by degradation in the crystal zone cannot diffuse into the hydrolysis medium on time, such low molecular weight substances will accumulate in the mesophase and might be catalytically degraded locally because of their acid properties [35]. This would result in bond cleavage in this region and might accelerate degradation at a faster rate of hydrolysis [36,37]. However, the increase in crystallinity did not lead to the accelerated degradation of PPDO. In summary, all consistent data demonstrated that isothermal annealing retarded the degradation of PPDO to some extent.

## 4. Conclusion

In this work, PPDO was compression-molded into bars, and the PPDO bars were subjected to isothermal annealing at various Ta for different ta. The synthesized PPDO underwent a process of melting, quenching, and annealing. The isothermal annealing provided sufficient temperature and time for the molecular chains to rearrange. During the annealing, the amorphous regions became locally ordered, and the arrangement of the molecular chains in the crystal became compact and regular, reducing the proportion of the amorphous phase. The PPDO (90 ℃, 2 h) reached maximum crystallinity (57.21%) and maximum tensile strength (41.1 MPa). The tensile strength was improved by 79.48% compared to untreated PPDO. The change of DSC double melting peak and recrystallization can indicate that the annealing process changed the internal structure. The XRD showed there was no crystal phase transition. With an increase in Ta, the FWHM decreased, the size of grains and crystallinity increased. Interestingly, the heat treatment process did not cause serious thermal degradation of PPDO. It was observed that isothermal annealing treatment delayed degradation for about a week. Consequently, PPDO can improve mechanical properties and delay degradation by annealing at 90 °C for 2 h. These properties make PPDO good candidates for use in medicine, such as bone or tissue fixation devices and drug delivery systems. The annealed PPDO has great potential for general materials in place of nonenvironmentally friendly materials, such as films, molded products, laminates, etc.
